# Climbing the 'ladder of intrusiveness': the Italian government's strategy to push the Covid-19 vaccination coverage further

**DOI:** 10.1007/s11077-023-09509-2

**Published:** 2023-05-14

**Authors:** Stefania Profeti, Federico Toth

**Affiliations:** Dipartimento di Scienze Politiche e Sociali, University of Bologna, Strada Maggiore 45, 40125 Bologna, Italy

**Keywords:** Policy instruments, Vaccination policy, COVID-19, Compliance, Italy

## Abstract

In all Western countries, the vaccination campaign against COVID-19 encountered some resistance. To overcome vaccine inertia and hesitancy, governments have used a variety of strategies and policy instruments. These instruments can be placed on a 'ladder of intrusiveness', starting from voluntary tools based on simple information and persuasion, through material incentives and disincentives of varying nature and magnitude, to highly coercive tools, such as lockdown for the unvaccinated and the introduction of the vaccination mandate. Italy's experience during the vaccination campaign against Covid provides an ideal observational point for starting to investigate this issue: not only was Italy among the top countries with the highest percentage of people vaccinated at the beginning of 2022, but—at least compared to other European countries—it was also one of the countries that had gradually introduced the most intrusive measures to increase vaccination compliance. In the article the different steps of the ‘intrusiveness ladder’ are presented, providing examples from various countries, and then tested on the Italian Covid-19 vaccination campaign between 2021 and the first months of 2022. For each phase of the campaign, the instrument mixes adopted by the Italian government are described, as well as the contextual conditions that led to their adoption. In the final section, an assessment of the composition and evolution of the Italian vaccination strategy is provided, based on the following criteria: legitimacy, feasibility, effectiveness, internal consistency and strategic coherence. Conclusions highlight the pragmatic approach adopted by the Italian government and underline the effects—both positive and negative—of scaling up the intrusiveness ladder.

## Introduction

In all Western countries, the vaccination campaign against COVID-19 encountered resistance, due to a variety of factors, as usually happens in vaccination policy (Dubé et al., 2013; Dubé et al., 2015; MacDonald, 2015; Betsch et al., 2018; Troiano & Nardi, 2021). To overcome vaccine inertia and hesitancy, and to increase the percentage of the population vaccinated, governments have used a variety of strategies and policy instruments. These instruments can be placed on a 'ladder of intrusiveness', starting from voluntary tools based on simple information and persuasion, through material incentives and disincentives of varying nature and magnitude, to highly coercive tools, such as lockdown for the unvaccinated and the introduction of the vaccination mandate.

Most of the literature on vaccine ethics (Childress et al., 2002; Giubilini, 2019; Saghai, 2014) and the advice given out by prominent international health organizations (Nuffield Council on Bioethics, 2007; WHO, 2021) converge in recommending that governments follow the *'principle of the least restrictive alternative*' when designing their vaccination campaigns, according to which one should climb the rungs of the intrusiveness ladder only to the extent that less intrusive measures have proven ineffective. However, as the literature on policy instruments has repeatedly pointed out, the selection of instruments does not only depend on technical considerations based on their effectiveness, but also has to reckon with other contextual and contingent factors, such as the (technical and political) feasibility (Salamon, 2002) and the perceived legitimacy of the proposed solutions (Capano & Lippi, 2017), as well as the characteristics of the target groups (Howlett, 2018). Moreover, taking a diachronic perspective, the transition from one instrument to another rarely results in a replacement, but rather in a process of layering in which new instruments are added to those previously adopted, which in turn can be recalibrated over time (Capano, 2019). The composite nature of instrument mixes and their changing nature over time call thus for attention to be paid to their internal consistence, in addition to their coherence with the aim they are intended to pursue (Rogge & Reichardt, 2016).

All of the above factors concur to complicate governments' choices of the most appropriate means to maximize vaccine compliance and may influence their propensity to climb to the top of the intrusiveness ladder or to stand on the first rungs. This holds especially true in the case of vaccination campaigns against COVID, given the vaccines' novelty, their short trial, the ever-changing nature of the virus (i.e. the problem to be solved) and the urgency of choices. In this respect, analysing governments' strategies when developing their instrument mixes to increase the number of anti-COVID vaccinations, the factors that led to these choices, and the effectiveness and consistency of the chosen solutions, may provide some original insights to the literature on both vaccine hesitancy and policy instruments. It is worth pointing out that, with few exceptions (Betsch et al., 2015; Attwell and Smith, 2018; Giubilini, 2019; McCoy, 2019; Paul & Loer, 2019; Attwell et al., 2021) the literature on vaccine hesitancy has had little interaction with that on policy instruments: these two strands of research have largely traveled in parallel. On the other hand, Italy's experience during the vaccination campaign against COVID provides an ideal observational point for starting to investigate these issues: not only was Italy among the top countries with the highest percentage of people vaccinated at the beginning of 2022, but—at least compared to other European countries—it was also one of the countries that had gradually introduced the most intrusive measures to increase vaccination compliance (Diaz Crego et al., 2022). Indeed, the government climbed the 'ladder of intrusiveness' over the months, starting with non-coercive instruments (based on information and moral suasion), steadily adding more constraining measures (recalibrating incentives and disincentives associated with the Green Pass), and finally arriving at the compulsory vaccination of the entire over-50 population.

Drawing upon these premises, the paper is structured as follows. In Section "The ladder of intrusiveness", the different steps of the ‘intrusiveness ladder’ are presented, providing examples from various countries. In Section "The COVID-19 vaccination campaign in Italy: climbing the ladder of intrusiveness", the Italian COVID-19 vaccination campaign between 2021 and the first months of 2022 are split into 5 successive phases, which have been singled out depending on the main alterations in the composition of the instrument mix, and their underlying rationale: for each phase, the instruments adopted by the Italian government are described, as well as the contextual conditions that led to their adoption. In Section "Discussion", we discuss Italy's vaccination strategy, assessing the composition and evolution of the instrument mix, based on the following criteria: legitimacy, feasibility, effectiveness, internal consistency and strategic coherence. In the final section, we draw conclusions from what has been argued above, highlighting the pragmatic approach adopted by the Draghi government and underlining the effects—both positive and negative—of this approach.

## The ladder of intrusiveness

Vaccines are one policy area where the issue of compliance, i.e. the matching between recipients' behaviour and decision makers' indications and expectations (Étienne, 2010), is of the utmost importance in decreeing the success or failure of public action, especially where non-compliance may entail unacceptable risks for the community (Edwards, 2006). In the case of vaccination campaigns aimed at broad-based immunisation, achieving the enrolment of a vast majority of citizens, while countering passive non-compliance and active resistance (McCoy, 2019), is undoubtedly one of governments' priority objectives. When the COVID-19 vaccination campaign began in late 2020 and early 2021, most Western countries found themselves in a situation where available vaccine supplies were limited, with a large part of the population wanting to get vaccinated as soon as possible. At that time, demand for vaccines exceeded supply (Evans & French, 2021). Consequently, rationing strategies had to be implemented, identifying priority categories to be vaccinated before others. Within a few months, however, in many countries the situation was reversed: there was a large supply of vaccines, but part of the population was reluctant to vaccinate.

In order to overcome this 'vaccination inertia' (which includes not only those who do not intend to vaccinate, but also those who—for various reasons—postpone vaccination), governments have adopted different strategies. These vaccination strategies made use of a variety of policy instruments, often in combination with each other, resulting in a plethora of approaches and solutions that need to be systematised before they can be analysed and assessed. To do so, of the many classifications of policy instruments offered in the literature (Acciai & Capano, 2021), for the sake of conceptual coherence it is convenient to resort to those that use as *fundamentum divisionis* the behavioral assumptions behind the selection of specific tools (Capano & Engeli, 2021), rather than focusing on those based on the type of resources mobilized by governments. Indeed, the basic problem for governments with vaccine hesitancy is to trigger citizens into adopting the desired behaviour. From this point of view, ideally drawing on typologies proposed by Schneider and Ingram (1990) and Doern and Phidd (1983), it is possible to place the instruments that governments have used to boost vaccine rollout along a "ladder of intrusiveness", according to how much freedom of choice they leave to recipients as to which behaviour to adopt, and what lever they try to tick to drive it. Let us try to review these instruments, giving some real examples.

### Plain information

The bottom rung of the ladder is mere information: the government collects and disseminates data, but does not direct citizens towards a particular course of action. Information is provided so that the citizen has the elements to evaluate and form an opinion. This has been done in all Western countries: through websites and other channels of communication, health authorities (the government, the Ministry of Health, the agencies responsible for regulating medicines) have provided information about the characteristics of anti-COVID vaccines and how to administer them.

### Hortatory tools

Going up the ladder of intrusiveness, we find what we might call 'hortatory tools' (Schneider & Ingram, 1990): the government explicitly takes a position, and recommends a specific behaviour. The aim is to convince citizens, with rational and/or emotional arguments, without imposing restrictions and without providing any kind of material incentive or disincentive (Giubilini, 2019).

Hortatory strategies can make use of a variety of arguments and 'motivational levers'. The arguments can be positive or negative, and with a different degree of emotionality. We can conceive of a climax of exhortation instruments that starts with *persuasion* (the advantages of a certain behaviour are presented), grows towards *exhortation* (civic and moral values are recalled), up to *admonition* (the aim is to 'frighten' citizens or arouse a sense of guilt in them).

Let's take a few examples from the communication campaigns used to promote the COVID-19 vaccine. The Canadian government's campaign, condensed into the slogans *'The vaccines protect you. The vaccines work. The vaccines are safe*', is an example of persuasion: the individual benefits of vaccination are presented, encouraging the population to get vaccinated. From persuasion we move on to exhortation when it relies on altruism and social responsibility. A slogan used by the Australian government was *'I protected myself for my community'*. US President Joe Biden called vaccination *'your patriotic duty'*.

Some national governments have gone further, using negative arguments, emphasizing the risks for the unvaccinated, focusing on fear and blame (Bardosh et al., 2022; Betsch et al., 2015). In these circumstances, we could speak of 'admonition'. Examples are the messages of the US CDC agency (“*Unvaccinated people are 17* × *more likely to be hospitalized with COVID-19*”), the advertising posters used in Russia (“*How many should die for you to get vaccinated?*”), the unfiltered public statement of French President Emmanuel Macron (“*I want to annoy the unvaccinated, as they endanger the life of others*”).

### Nudge

The third rung of the intrusiveness ladder is represented by nudging strategies (Thaler & Sunstein, 2008). Also in this case, the government has a preferred option, but this preference is not necessarily made explicit, or in any case is communicated less directly and more subtly than with hortatory tools. For this reason, nudge is on a step above the latter on the scale of intrusiveness, as it can influence the behaviour of individuals more or less outside their awareness (Marchiori et al., 2017). By nudging, the government—following a typical 'libertarian paternalist' approach—gently drives towards preferred behaviour through mechanisms that alter the individual choice architecture, while not prohibiting any options or introducing any significant economic benefits (Thaler & Sunstein, 2008). Nudges deal with the way in which choice options come pre-structured (e.g. by defining 'default' rules to exploit status quo biases), how they are laid out (e.g. by making preferred options more visible, or more easily accessible than others), as well as informing people of their 'popularity' (e.g. by telling that most people—or renowned people—are engaged in a certain behaviour). But nudging can also be about warning and reminders, as well as simplification tools to ease people's daily lives and lessen the burden associated with some choices (such as by reducing red tape and paperwork requirements) (Sunstein, 2014).

In the specific case of the COVID-19 vaccination campaign, the most frequently used nudging mechanism was the invitation to vaccinate "because many others are doing it". This motivational lever is based on the assumption that individuals are generally inclined to 'follow the herd'. The communication campaign launched by the English NHS in April 2021, with the slogan *'Join the million already vaccinated'*, fits perfectly into this picture. The same principle lies behind the generalized practice of daily broadcasting figures regarding the number of newly vaccinated, as well as the common and widespread recourse to public communication campaigns centred on celebrity endorsements. Either way, though less frequently, also default and simplification nudges had been used worldwide: for example, in the Swedish region of Uppsala letters with pre-scheduled vaccination appointments were sent to selected demographic groups in July 2021, with them being given the burden of opting out (Bonander et al., 2022). Around the same period, the French government changed the rule that second jabs must be given same place as the first, allowing holidaymakers to have access to COVID vaccines wherever they were in the country, including beaches and tourist spots.^1^ Walk-in vaccination schemes or sessions were envisaged in various countries, from the UK to Bangladesh (Faruk & Al Quddus, 2023). In a similar vein other countries, such as Switzerland, the UK and Ireland, allowed local community pharmacists to administer the COVID-19 vaccine, assuming that both proximity and trust would have a positive effect on people's willingness to vaccinate (Paudyal et al., 2021).

### Positive incentives

Moving further up the ladder of intrusiveness, one moves—as Vedung (1998) puts it—from 'sermons' to 'carrots'. The latter strategy consists of providing positive material incentives. By doing so, the logic through which citizens' compliance is pursued changes: informational and hortatory instruments aim at convincing individuals; the carrot strategy, on the contrary, assumes that individuals go along with the preferences of policy-makers not because they are intrinsically convinced, but because they find it convenient to do so.

In the case of COVID vaccination, different national and sub-national governments have made use of a variety of incentives. Incentives may consist of cash or in-kind premiums envisaged for the vaccinated. Rewards may be of limited economic value, such as free public transport tickets, free taxi rides or food discount and vouchers for the vaccinated, as was done in the UK through a deal between the government and some private companies.^2^ Discounts in shops, free meals, free admission to parks and tourist attractions were also offered in many countries (Tinari & Riva, 2021). But premiums can grow in value. In several US states (including the 'Vax-A-Million Lottery' in Ohio) and Canadian provinces, as well as in Poland and Russia, lotteries (with rich prizes) have been organised for individuals who have been vaccinated (Fuller et al., 2022; Dubé et al., 2022; Kuznetsova et al. 2022). Many US states went so far as to offer a cash reward after the White House called on states and local governments to directly pay $100 to anyone willing to get a first dose (Oza, 2021).

### Negative incentives

In addition to positive incentives, policy-makers may also consider negative incentives. The Singapore government, for example, stipulated in December 2021 that those 'unvaccinated by choice' would have to pay out-of-pocket for any COVID-19-related medical treatment (Vogel & Duong, 2022).

It is important to note that the shift from positive to negative incentives usually implies an increase in the intrusiveness rate (Giubilini, 2019; Nuffield Council on Bioethics, 2007). Incentives and disincentives are perceived differently by citizens. In the positive reward there is the message "if you behave in a certain way you get a bonus, but you are free to behave as you like". A disincentive can be perceived as a penalty, or as an action the government wants to stigmatize.

### Personal restrictions

Personal restrictions on performing certain activities also fall into the category of negative incentives. Personal restrictions are typically regulatory instruments with which the government ends up restricting individual freedom. Only those who have been vaccinated (or are immune to COVID-19 because they have previously contracted the disease) can carry out certain activities. This implies, by reversing the perspective, that those who are not vaccinated cannot enter certain places or freely carry out certain activities. In many European countries, for example, a 'vaccine pass' or 'Corona vaccination certification' has been introduced: those who do not have this certification are restricted in their travel and movement, access to certain public places, participation in sporting, recreational or cultural events, etc. (Kuznetsova et al., 2022; Oliu-Barton et al., 2022).

The most extreme form of restriction on certain activities is lockdown for the unvaccinated, such as those introduced in Austria, Slovakia and Germany at the end of 2021 (Redlin, 2022).

### Mandates

The top rung of the intrusiveness ladder, corresponding to the highest level of government coercion, is compulsory vaccination. This strategy nullifies the individual's freedom of choice. In some countries, vaccination requirements have been restricted to specific categories (defined by profession, age, or health condition). Many national governments, for example, have introduced the *'no jab, no job*' rule, whereby compulsory vaccination is targeted at certain categories of workers (Stokel-Walker, 2021). In many countries—including France and Germany – compulsory vaccination has been introduced for health and/or school staff (Bardosh et al., 2022). In Greece, the over-60 s population has been obliged to be vaccinated since January 2022 (Charrier et al., 2022).

Finally, there are countries—including Indonesia (from February 2021), Ecuador (from December 2021) and Austria (from February 2022)—where the vaccination requirement has been extended to the entire adult population (Mtimkulu-Eyde et al., 2022).

## The COVID-19 vaccination campaign in Italy: climbing the ladder of intrusiveness

In this section, we review the COVID-19 vaccination campaign in Italy, pinpointing the stages based on the policy instruments used by the government to maximise citizen compliance. For each phase the policy mix adopted by the Italian government to boost the vaccine rollout is identified, contextualizing it with respect to the specific environmental conditions of the period (e.g. availability of vaccines, trends in the number of contagions, political and governmental dynamics, choices made by other European governments, etc.).

### The first phase—hunting the vaccines (Jan–Feb 2021)

As elsewhere in the EU, in Italy the vaccination campaign against COVID-19 symbolically started on 27 December 2020. Italy's vaccination plan, approved on 2 January 2021, outlined the essential elements of the campaign's organisation, identifying phases based on priority target groups for vaccine administration. Both the time sequence and the prioritisation of the target groups followed the recommendations that the EU had made to the member states in October 2021, with a number of phases to be adapted according to vaccine availability. The first and second phases were to be dedicated to high-risk groups on account of their job (healthcare workers), their health status (frail people, especially elderly living in residential facilities) and age (first people over 80, then people between 60 and 79 years old). Based on the indications of the international drug regulatory agencies, RNA vaccines (Moderna and Pfizer) should preferably be targeted to these categories, while viral vector vaccines (such as Astrazeneca) were addressed to people between 18 and 55 and to over 55 in good health.

However, in the first quarter of 2021, the doses of RNA vaccine actually available were around 44% lower than expected, particularly when looking at the Moderna vaccine targeting the elderly and frail. In this context of scarce resources, the problem for the government was not so much to tackle vaccine hesitancy as to manage demand and create a favourable mood for vaccination ahead of the next steps. At this stage, the government's strategy therefore focused almost exclusively on non-intrusive, hortatory tools: alongside the plain information channels provided by the Ministry of Health and the Regions on the characteristics of the vaccines available and on the vaccination trend (which remain in place throughout all stages of the campaign), there were a few institutional communication initiatives that called for vaccination by focusing on an emotional register and highlighting the civil and caring responsibility towards others. Two examples of this communication strategy are the 'primroses' project, i.e. the provision of flower-shaped vaccination pavilions designed to evoke the waking up from the pandemic, and a series of TV spots designed and made by the renowned Italian director Gabriele Tornatore that portray vaccination as an act of love towards one' s beloved ones.

Little emphasis was placed instead on removing logistical barriers to accessing vaccination points, despite the fact that this could be a potential compliance obstacle especially for one of the early priority groups, the over 80 s: indeed, the national vaccination plan published in January provided that, in the first phase, vaccinations would be carried out in medical facilities and hospitals and in not clearly specified mobile units, giving the regions plenty of room for manoeuvre in deciding how to administer anti-COVID jabs. As a result, once phase 1 was still underway, some regions^3^ began focussing on non-priority categories (such as lawyers and journalists, to mention a few) given the difficulties of reaching the over 80 s. By the end of February 2021, the proportions in the most-at-risk age categories who had received a first dose varied considerably from one region to another (Profeti, 2022), as infections began to climb again in the middle of the virus' third wave. Faced with a full-on vaccination hunt, the press started to stigmatise the phenomenon of vaccine 'scoundrels', i.e. those who skipped the queue by not waiting their turn, to the detriment of the most vulnerable groups.

### The second phase—getting ready for mass vaccination (March–May 2021)

The Italian vaccination campaign reached a first turning point in March 2021, following an internal crisis in the majority that supported the centre-left government led by Giuseppe Conte. A new grand coalition government was formed in February, with the support of almost all the parties represented in Parliament (with the exception of the right-wing political force Fratelli d'Italia—FdI). On 1st March, the new Prime Minister, Mario Draghi, replaced the previous extraordinary Commissioner for the emergency, Domenico Arcuri, with army general Francesco Paolo Figliuolo, who immediately took office and proceeded to drastically reorganise the vaccination campaign, starting with the logistics. First, the number of vaccination points was expanded by using existing and available public and private facilities, such as gyms, sports halls, barracks, etc., alongside drive-throughs staffed by the military. In the meantime (March 7), a national protocol agreement was reached with GPs, notably to ease vaccinations for the elderly. Furthermore, in April more stringent coordination and control mechanisms were put in place by the central government with regard to the organisational choices made by the regions, the latter being required to meet weekly vaccination targets and to follow the administering priorities more strictly. Finally, in the expectation that vaccine supplies were to be less spotty from mid-March, more explicit and more ambitious targets were set by the Commissioner. These were to deliver at least 500,000 doses per day by the end of April, and to achieve coverage (60% of the population by the end of July, 70% by the end of August and 80% by the middle of September) sufficient to ensure herd immunity ahead of autumn.

Besides these rather sharp organisational adjustments, which mostly served to get the machine ready for the start of the mass roll-out at the beginning of June, the Italian government introduced two regulatory instruments to promote compliance that ranked higher on the ladder of intrusiveness: first, vaccination was made compulsory for health professionals as of 1 April (Italy being the first country in Europe), even though about 92% of doctors and nurses had already received their first dose and 76% had already completed their vaccination cycle on that date^4^; second, following on from the digital certificates issued by the European Union in March 2021 as a means of combining citizens' mobility with limiting disease, Decree N. 52/2021 (April) introduced the so called Green Pass which became necessary for travelling between regions and for attending public events where social distancing was impossible to maintain (i.e. concerts or sporting events). In this initial form, the obligation to carry a Green Pass—issued not only to the vaccinated or those recovered from COVID, but also to those having a negative result from a test taken in the previous 48 h—was more akin to an incentive (which can be both seen as positive for those who choose to vaccinate, as it offers the possibility of resuming certain social activities suspended during the pandemic, and as negative for the unvaccinated) than a real coercive measure, as it was designed to make it somehow inconvenient not to get vaccinated, but not actually to prohibit anything (Campanozzi et al., 2022; Stefanizzi et al., 2022). On the other hand, some scholars also interpreted the Italian Green Pass as a kind of nudge, as the opt-out condition—i.e. taking a swab every 48 h—would have been much more costly (in terms of time and money) than receiving the vaccine (Reno et al., 2022).

### The third phase—reaching the wait-awhiles (June–July 2021)

**Fig. 1:**
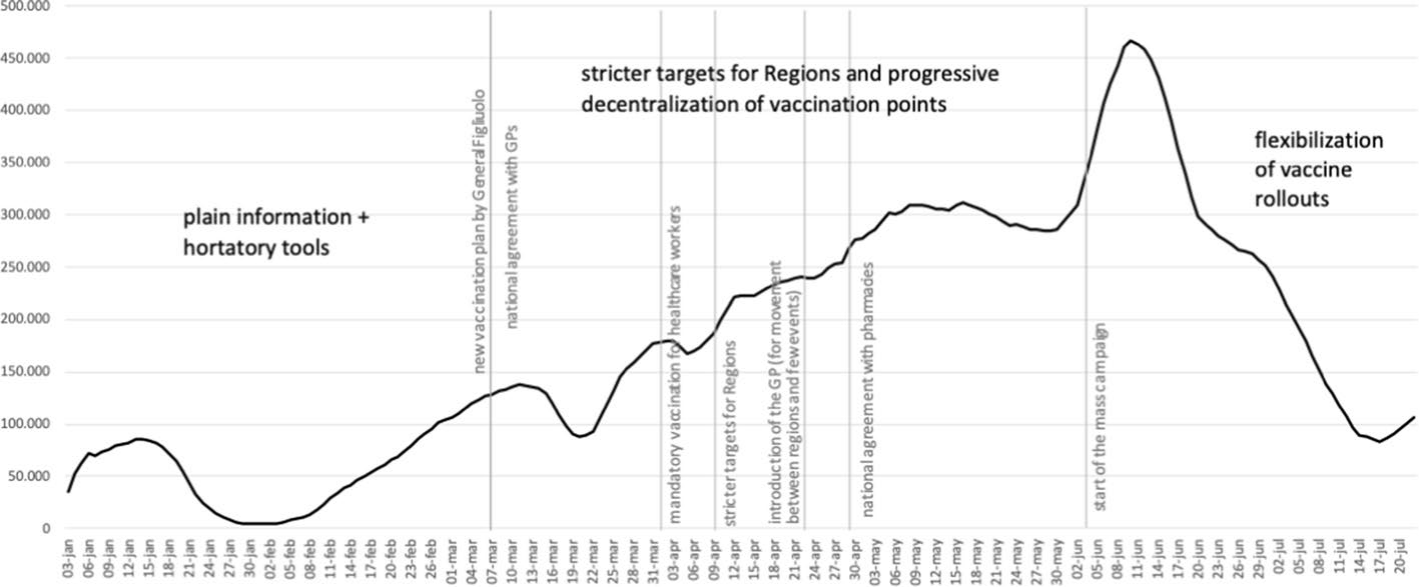
Trend in first doses (seven-day moving average) between the 1st and 3rd phase. Source: authors’ elaboration of data at: https://github.com/italia/covid19-opendata-vaccini

While from March onwards the quantity of vaccine doses available to the Italian government grew steadily until it reached its peak between May and June (as did the number of first doses administered daily), as the summer was approaching the number of new vaccinated people tended to stabilize, and from mid-June onwards it began to drop dramatically (Fig. 1). Indeed, the start of the mass vaccination campaign aimed at all sections of the population (June 3) coincided with a slowdown in the number of infections which, coupled with the onset of the warm season, may have led to a decline in the perception of individual risk. In addition to that, the braking effect of the 'Astrazeneca affair' on the progress of vaccine uptakes from mid-March 2021 onwards should be mentioned: as some studies have shown, the Italian government's decision to suspend the administration of Astrazeneca doses for four days as a precautionary measure following alleged (but never confirmed) connections between that vaccine and rare blood clots not only caused 20% of those who had booked to refuse (and cancel) that vaccine in the short term (Barello et al., 2022), but also resulted in a significant reduction in Astrazeneca injections that persisted weeks after authorities reassured the public about the vaccine’s safety (Deiana et al., 2022, 1267).

Against this backdrop, and once the group of people who were willing to vaccinate immediately had been exhausted, the government was then faced with the challenge of convincing those who, in the literature, came to be known as 'wait awhiles'; that is, people who, for various reasons (e.g. because they wish to have more data, easier access, a different brand of vaccine, or because they see the virus as barely dangerous), hesitate and put off being vaccinated (Carlson et al., 2022). Unlike other countries (European and non-European), in Italy there has been almost no recourse to positive incentives (monetary or in kind) to get this target group on board. Rather, the strategy pursued by the Italian government had been to remove as far as possible burdens that might discourage or make it inconvenient to go for the vaccine, through a progressive flexibility in organization: not only the decentralisation of vaccination points, which was decided by the Draghi government under the reorganisation of the Italian vaccination plan, but also other more nudge-like measures such as the possibility of taking the vaccine at trusted (and proximity) venues, such as local pharmacies and paediatricians, the opportunity to book second doses outside one's region of residence for holidays, reservation-free open days and mobile vaccination points on beaches, in airports, etc.

On the other hand, the government tried to harness the Italians' desire to return to normality in the run-up to the holidays. First, in terms of hortatory tools, the focus of institutional communication shifted from a call for respect and care for others to a wish to resume a full social life: an example of this strategy is the TV spot *'Riprendiamoci il gusto del futuro*' ('Let's take back the taste of the future'), launched by the government in June, in which famous sports and entertainment personalities invited Italians to get vaccinated. Then, on the regulatory side, the Green Pass was also extended to the key activities of the summer social life: indeed, on 23 July 2021, Decree N. 105 provided that from 6 August the Green Pass would be necessary in order to gain access to indoor restaurants and bars as well as other social settings such as theatres, swimming pools, festivals, etc. Prime Minister Draghi, in his announcement of the new Green Pass during a press conference, portrayed it as “a measure that brings you serenity”, at the same time stating that “the call not to vaccinate is a call to die”.^5^

Looking at the data, the measures introduced during this period produced some positive effects on the number of new vaccinated. The amount of first doses administered daily began to grow again in July, albeit not straightforwardly (Fig. 1), and the target of 60% of the population being vaccinated by the end of July was achieved (Profeti, 2022). In some TV interviews when the new Green Pass came into force, Special Commissioner Figliuolo made enthusiastic remarks on its effects, claiming a growth in first-dose bookings of between 15 and 200%, varying by region.^6^ The number of newly vaccinated was higher among the younger age group between 18 and 24 (Mills & Rüttenauer, 2021), probably because they were more sensitive to the possibility of enjoying an active summer social life.

On the other hand, the further restrictions associated with the Green Pass triggered the first protests in the streets by the so called "no vax", who framed them as liberticidal policies,^7^ and generated some rifts within the government majority. Among the parties that were part of the grand coalition, only the Democratic Party (PD), Italia Viva (IV) and Forza Italia (FI) had a compact favorable position on the Green Pass (Russo & Valbruzzi, 2022). The Five Star Movement (M5S), with an overall parliamentary weight of 26.7%, had been strongly against compulsory vaccination in the pre-pandemic phase (Cadeddu et al., 2020; Casula & Toth, 2021), and with respect to the Green Pass appeared divided, with some of its members calling for mandatory certification limited to stadiums and discos, but not for bars and restaurants. The League (with an overall parliamentary weight of 21.8%) was certainly the government partner most hostile to introducing more intrusive measures: in fact, prior to its participation in the Draghi government, they shared with FdI very similar positions on Conte's government's handling of the pandemic, criticizing him for being too intrusive in his approach (Albertazzi et al., 2021). Moreover, like the M5S, the League had strongly opposed a compulsory vaccination requirement for children over the previous years (Cadeddu et al., 2020; Casula & Toth, 2021). While FdI, that remained in opposition to the Draghi government, had been able to keep an outright negative stance on vaccines and green passes, thereby gaining a certain issue ownership, the League was instead more ambiguous, struggling between justifying its support to the government and avoiding consigning part of its electorate to a party like FdI, targeted at the same electoral pool (Russo & Valbruzzi, 2022). That accounts for some of the tensions recorded as early as July 2021 between the party's national leader, very much hostile to the expansion of the Green Pass, and the League ministers more aligned with government policy decisions.

However, compared to what has been observed in the literature (Dobus & Tosun, 2021), the politicization of COVID vaccines within the Draghi government only partly matches self-reported electorate support: while the majority of Italians who expressed opposition to the Green Pass voted for FdI (41% of party voters), the vast majority of voters for the other parties were favorable to the certificate (ranging from 94% of PD voters to 79% of FI voters) (Russo & Valbruzzi, 2022).

### The fourth phase—making hesitancy costly (August–October 2021)

**Fig. 2:**
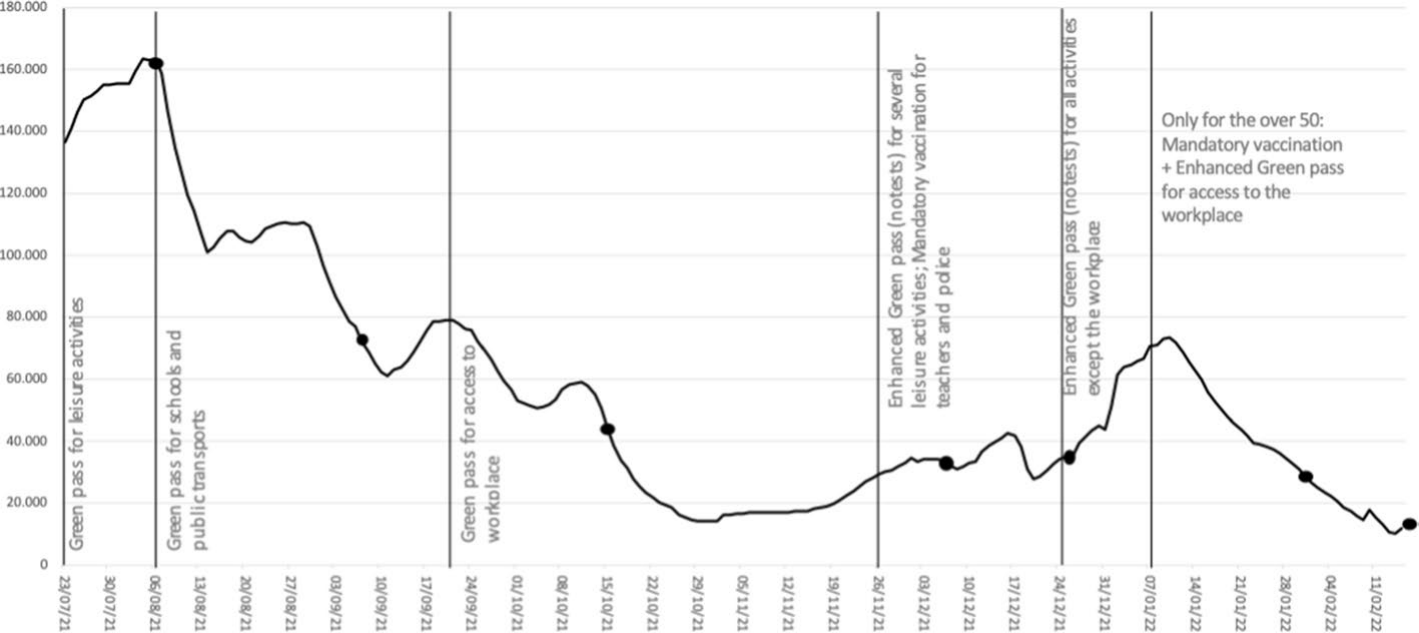
Trend in first doses (seven-day moving average) after Green Pass and its re-calibration. Lines: entry into force of the legislation. Dots: Measures begin to be effective. Source: authors’ elaboration of data at: https://github.com/italia/covid19-opendata-vaccini

In the framework of a sluggish vaccination trend (Fig. 2), a series of critical issues emerged between August and October 2021 which, taken together, led to a necessary review of the government's vaccination campaign strategy.

Firstly, schools were scheduled to reopen in September, making it difficult to maintain physical distance indoors among children (not yet eligible for vaccination) and teens. The issue of guaranteeing school attendance had occupied a prominent place in the public debate since the first phase of the pandemic emergency (Pavolini et al., 2021), but at the beginning of August 2021 about 15% of teachers, as well as more than 63% of teens, had not yet received any vaccine dose.^8^ On the other hand, the spread of new variants of the virus (Delta since July, and Omicron since October) characterized by higher transmissibility and a greater capacity to bypass vaccines, made the government set aside the hypothesis of achieving herd immunity by following the vaccination targets originally envisaged (namely 80% by middle September). This did not mean abandoning efforts to increase the number of vaccinated persons; rather, although expectations of the vaccination campaign's effect on virus transmission had dwindled, the government continued to point to vaccines as the major tool to mitigate the individual risks associated with the disease and as a means of reducing pressure on hospitals, especially in view of the fall season and the possibility of new waves of infection. Indeed, as reported by the GIMBE Foundation, as of the end of July 2021, still only 88.5% of the over-60 s (i.e. the group most at risk against the virus and for which the government's vaccination plan had from the outset intended to give priority) had received the first dose of the vaccine, with a minimal national weekly increase (+ 0.5%) despite the actions taken in recent months.^9^

Against this background, most of the government's efforts focused on strengthening the Green Pass instrument, which, while remaining formally distinct from mandatory vaccination, sharply climbed the ladder of intrusiveness so as to make non-vaccination a very costly option for most citizens. Indeed, in August Decree N. 111/21 extended the Green Pass obligation to all those employed in education, to university students willing to attend in-person classes, and to those using long-distance public transport; in September Decree N. 127/21 extended the obligation to all public- and private-sector workers from 15 October, providing that workers who were not compliant were to be considered as absent without leave, meaning that they would lose pay. Also with respect to these provisions, the governing majority failed to look cohesive, with the League party repeatedly voting in parliament for amendments proposed by the opposition.

**Fig. 3:**
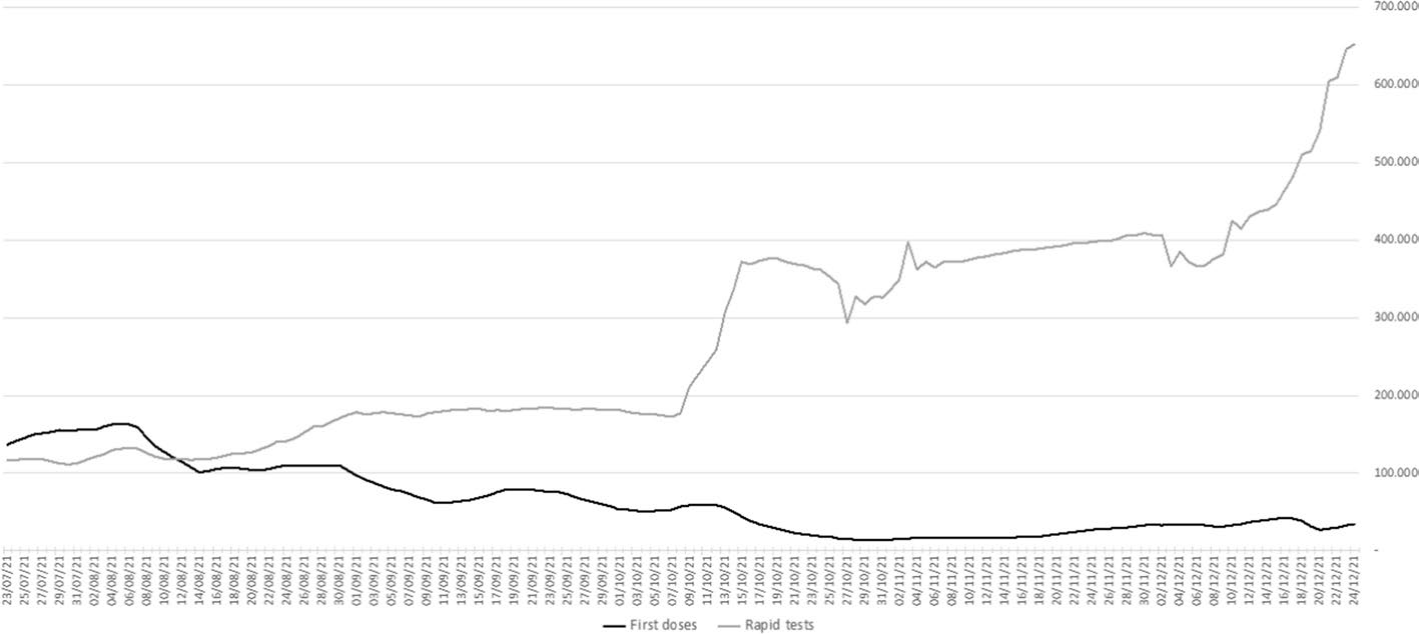
First doses VS. rapid saliva tests (seven-day moving average). Source: authors’ elaboration of data at: https://github.com/italia/covid19-opendata-vaccini

These measures, while allowing the opt-out from the vaccine through the possibility of showing a negative test carried out within the last 48 h, were aimed at making that option burdensome in economic, time and psychological terms. After the announcement of the mandatory Green Pass for access to workplaces in mid-September 2021, an increase in first doses was indeed recorded, particularly in the working-age population between 50 and 59 (with a weekly increase of 51.9% in new first doses compared to the first two weeks of September) and in the 40–49 group (with an increase in about 44%).^10^ However, like in other countries that adopted a similar policy of mandatory COVID certificates, the upsurge of new vaccinations was confined to the week following the announcement of the measure and the few days after its entry into force, with the momentum gradually wearing off (Mills & Rüttenauer, 2021): as a matter of fact, the impact of the Green Pass on the numbers of new vaccinations seemed to decline over time, while there was an exponential growth in the number of rapid antigen tests carried out, as shown in Fig. 3. We can, therefore, assume that—at least in Italy—people who were most reluctant to vaccinate against COVID-19 have been ready to bear the burden of opting out, and to test every two or three days so as to obtain the Green Pass without having a shot.

### The fifth phase—fighting reluctance with coercion (November 2021–January 2022)

In November, a fourth wave of infections resumed across Europe, and Italy was no exception. Towards mid-November, the number of daily infections rose to over ten thousand, and then ten times that number in December. The rates of hospitalization and intensive care also began to climb, although—thanks to vaccines—they remained much lower than in the early stages of the pandemic. New vaccinations, on the other hand, continued to grow very slowly: even though in mid-November 2021 about 80% of the population had completed the vaccination cycle, and 30% of these had already received the booster dose,^11^ surveys showed the consolidation of a hard core of reluctant people, recalcitrant to any kind of persuasion or incentive (whether positive or negative), estimated at slightly more than 10% (Moroni & Vezzoni, 2022). Vaccine hesitancy was higher in the 30–39 and 40–49 cohorts, both with around 16% unvaccinated, and declined as age increased, with only 3% unvaccinated among the over-80 s.^12^

Besides a partial reorientation of institutional communication towards nudging, with spots emphasizing that most Italians have had their vaccine and that it represented a lever for the country's recovery, the path chosen by the Italian government to combat that persistent resistance was first to further tighten the Green Pass: indeed, Decree N. 172 of 26 November provided for a ‘enhanced Green Pass’ available only to the fully vaccinated and those having recovered from COVID. The ‘basic’ Green Pass, obtainable with nothing more than a negative test, would only give access to the workplace, public transport, as well as to a limited number of facilities. A further strengthening of the Green Pass took place during the Christmas period, when there was an explosion of contagions due to the spread of the Omicron variant and the collapse in the availability of antigenic tests, the demand for which had continued to soar: with Decree N. 221 of 24 December 2021, the enhanced Green Pass became necessary for all activities with the sole exception of access to workplaces. The final squeeze which placed the Green Pass among the most high-intrusive instruments came in January 2022, when Decree N. 1/2022 provided for the enhanced Green Pass obligation for all workers aged over 50 to access the workplace too, with the employer being responsible for control and the penalty of suspension without pay and/or fines of €600 to €1,500 in case of non-compliance.

Following the moves towards compulsory vaccination in other European countries (such as Austria and Greece), the same Decree also provided for mandatory vaccination (from 1 February) for all people over 50 years of age, irrespective of being employed or not. With this decision, for the first time, compulsory vaccination was no longer linked to the criterion of high-risk professions (such as healthcare workers, as well as teachers and police—required to be vaccinated as of the end of November 2021), but to that of age. Although the government justified its choice on the grounds that the over-50 s were more likely to contract serious forms of the virus, the decision seems rather to be the result of a compromise among the parties in government, some of which (the League in the lead) were strongly opposed to a universal obligation. The same interpretation may be valid for the fines associated with non-compliance, which are considerably lower than in other European countries (namely €100 one-off compared with €100 a month in Greece and €600 every three months in Austria).

**Fig. 4:**
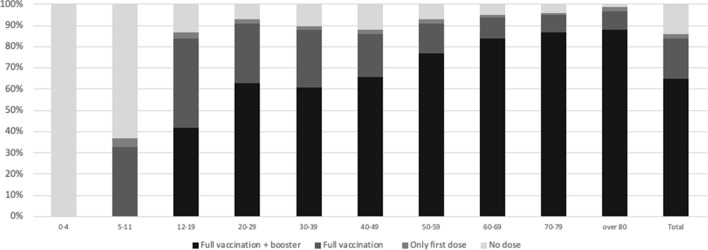
Vaccination uptake by age group, March 2022. Source: authors’ elaboration of data at: https://github.com/italia/covid19-opendata-vaccini

According to some estimates made at the beginning of February 2022, the introduction of these more stringent measures resulted in 1/3 of the over-50 s still not vaccinated taking their first dose within a month.^13^ However, the number of new vaccinations began to fall again from mid-February onwards, falling to almost zero in the next few weeks. In March 2022, when the state of pandemic emergency was officially called off, 84% of the Italian population had completed the vaccination cycle with at least two doses, and about 65% had received a booster third dose, albeit with considerable variations depending on the age group. The unvaccinated stood at an average of 14.5%, being concentrated in the middle age groups as well as among children and teenagers for whom, however, the vaccination campaign had started later (Fig. 4).

## Discussion

Let us now try to read the evolution of the instrument mix discussed so far with the help of some consolidated analytical categories provided by the literature on policy tools. As Salamon (2002) points out, besides the classical economic criteria for assessing public intervention (namely effectiveness, efficiency and equity), the policy literature suggests two further dimensions, manageability and legitimacy, that have to do with the technical and political feasibility of policy solutions, respectively. Indeed, «the choice of policy instruments is the result of the way in which decision makers combine the search for effectiveness with that for sense-making» (Capano & Lippi, 2017, 293), meaning that the selected tools must not only demonstrate effective intervention in the problem they are intended to solve, but also be technically feasible and socially acceptable (that is, perceived as legitimate) in the eyes of policy targets (Howlett, 2018). Moreover, in vaccines as well as other policies, governments often do not resort to a single type of instrument, but rather opt for mixed policy portfolios whose composite nature and changes over time call for attention to be paid to the consistency of the instrument mix (i.e. the absence of internal contradictions) and the coherence of the policy strategy (i.e. the alignment with the objective they intend to pursue) (Rogge & Reichardt, 2016). Hence, *legitimacy, feasibility, effectiveness, consistency* and *coherence* are the criteria against which we assess the instruments used by the Italian government to run the vaccination campaign and increase compliance.

### Legitimacy

Looking at the steps illustrated in Section "The COVID-19 vaccination campaign in Italy: climbing the ladder of intrusiveness", the Italian government seems to have adhered to the golden rule of the "*principle of least restrictive alternative*". This strategy is justified by the following assumption: the least intrusive instruments are not necessarily less costly for the government, but they are the ones generally considered more legitimate and more 'politically acceptable' by most of the population. However, the perception of the legitimacy of policy instruments is not the same in all policy areas and national contexts: in fact, it depends not only on the objective degree of coercion, but even more on the congruence with the legacy of previously adopted solutions, which in turn shape the perception of what is considered acceptable in the eyes of the recipients. What we mean—quite simply—is that if people are used to certain intrusive instruments, they may end up considering them more acceptable and less illegitimate than they are perceived in other contexts.

This may be as true for policy targets as it is for decision-makers. For example, unlike what happens in Anglo-Saxon countries in the field of health promotion policies (and in particular in the field of vaccination policies), in Italy there is little familiarity with monetary incentives. Most of the population would consider bizarre, perhaps even inappropriate the decision to offer cash rewards to those who vaccinate (or who donate blood, or who undergo a screening campaign). On the other hand, Italy has a long legacy of underutilisation of persuasive communications in the domain of vaccines: in fact, instead of employing communications to reassure the hesitant, Italian governments have usually focused "on systemic and delivery issues, until it was too late to do anything except make vaccinations mandatory (using modulation)” (Attwell et al., 2021, 457), as for example was recently the case for routine childhood vaccinations (Casula & Toth, 2019, 2021). These two aspects, both linked to the dimension of legitimacy, perfectly fit some of the choices made by the Italian government in devising the instruments to achieve COVID vaccination compliance, such as the very bland—albeit constant—recourse to communication-type instruments, and the near total absence of material rewards amongst the positive incentives put in place between the second and third phase of the campaign. Rather, the Draghi government has progressively climbed the ladder of intrusiveness, skipping the instruments with which Italians are less familiar, and preferring those previously experienced in the field of health and vaccination policies.

However, in spite of a certain tradition of childhood mandatory vaccination (Cadeddu et al., 2020), and although Italians in favour of mandatory mass COVID vaccination were estimated at around 60% according to various polls conducted between April and December 2021,^14^ the Draghi government used the highest level of coercion in a very selective way: first by introducing it only for the professional categories most exposed to risk, and not until early 2022 to a specific age cohort (the over-50 s).

This choice can be partly understood from an external legitimacy perspective: no European country, at least until December 2021, had in fact envisaged compulsory vaccination against COVID (except for a few at-risk categories such as health personnel, e.g. in France and Germany), nor had that issue ever been discussed within the EU institutions. The Italian government's decision to introduce compulsory vaccination for the over 50 s came only after the President of the European Commission Ursula Von der Leyen broke the taboo at the beginning of December, placing compulsory vaccination among the options to be discussed to halt the new wave of pandemics throughout Europe, and after its adoption was announced in some countries, such as Greece and Austria. In other words, what had already been decided by other national governments has plausibly pushed the Draghi government to do the same (if others do it, we can do it too).

### Feasibility

On the other hand, mandatory vaccination definitely raised more feasibility issues, both technically and politically, than the Green Pass (even in its enhanced version): on a technical level, whereas in the case of the Green Pass the entire burden of controls is placed on the business operators or employers, in the case of compulsory mass vaccination it would have been up to the public authorities, which would have required a substantial exchange of information between local health authorities (which would have had to report non-vaccinated persons) and the revenue agency (which would have had to impose sanctions), thus entailing very high organizational costs. Besides, on a political level, while the only party within the governing majority opposed to the Green Pass was the League, with regard to compulsory vaccination even the 5SM (i.e. the political force with the highest number of representatives in parliament) was divided, as it had assumed a clearly contrary stance in its campaign for the 2018 general election (Cadeddu et al., 2020; Casula & Toth, 2019). In short, against these conditions, the choice of climbing the ladder of intrusiveness through the progressive tightening of the Green Pass regulation, via incremental adjustments as and when the vaccine rollout fell short, probably looked like the more manageable option. The measures introduced during the fourth phase, which extended the Green Pass obligation from leisure activities to the workplace, actually occurred each time at the same time as a slowdown in the administration of first doses, yet showing a gradual decline in their effectiveness to get the campaign back on track.

### Effectiveness

Our analysis looked at the diachronic development of the Italian government's toolbox to boost vaccine uptake, paralleling it with vaccination trends using simple time series. It is thus beyond the scope of this article to assess conclusively how effective the various measures taken by the Italian government have been to persuade hesitant people to vaccinate. As regards the Green Pass, that is by far the measure the Italian government has bet most on, some information is provided by a few statistical studies based on counterfactual analyses: they estimate that the basic Green Pass accounted for 9.7% of people getting vaccinated in 2021 (Oliu-Barton et al., 2022), but they also show that the positive effect on new vaccinations occurred mainly between the announcement of the measure and its entry into force, declining rapidly thereafter (Mills & Rüttenauer, 2021).

Of course, the drop in the number of new first doses is partly physiological: the more people who have already been vaccinated, the smaller the number of those who have yet to do so. Either way, the latter are conceivably those who are more hesitant and less inclined to get vaccinated, especially if they are given an alternative to obtain a Green Pass, albeit a pricey one (the swab). Indeed, our analysis came to show that from the moment the Green Pass obligation was extended to the workplace, instead of a sharp increase in first doses there was a sudden surge in rapid saliva tests (Fig. 3). The attempt to further burden opt-outs (i.e. the swab) by extending mandatory certification to key activities in daily life (i.e. working) appears therefore to have not worked to win over die-hard hesitants.

The more intrusive measures taken in the fifth phase (the enhanced Green Pass only for the vaccinated, later also made compulsory for access to workplace for the over-50 s along with the mandate) can thus be understood as a response to the declining effectiveness of the basic Green Pass. Indeed, such more intrusive measures proved somewhat effective—though not dramatic—in getting the first doses up, especially around the Christmas holiday period. Limited to the over-50 s (i.e. the target group for the most intrusive provisions), they are estimated to have resulted in about 450,000 additional new first doses between 7 January and 15 February 2022 (about 15% of the unvaccinated in that age group), only to see them drop again to almost zero in March.^15^

### Consistency

The remodelling of the Green Pass in a restrictive key led to some flaws in the internal consistency of the instrument mix: in fact, the enhanced Green Pass, especially after it was made compulsory for access to workplaces for the over-50 s, began to drift away from incentives and move closer to coercion, imposing fines (from 600 to 1500 euros, plus loss of wages) difficult to sustain and much heavier than those associated with the outright compulsory vaccination (100 euros one-time). There has been, in short, a kind of "nominal inversion" of the two instruments: compulsory vaccination for the over-50 s is shaped as a polite mandate, akin to a negative incentive in practice, while the Green Pass, originally conceived as an incentive, takes the form of a disguised obligation. Likewise, the incremental layering of policy instruments (basic Green Pass, enhanced Green Pass, and mandatory vaccination) and their application to different targets (under 50 vs. over 50; workers vs. non-workers) yields undeniable disparities in treatment between individuals belonging to the same category (age or employment), with potentially detrimental effects on equity.

### Coherence

On the other hand, during the fifth phase some flaws regarding the coherence of the policy strategy—i.e. the coherence of the set of instruments adopted given the purpose they are meant to serve (Capano & Howlett, 2020)—also emerged. When introduced in summer, against a background of limited spread of infection, the aim of the Green Pass had clearly been to increase adherence to the vaccination campaign on the assumption that vaccines would be effective in preventing contagion. Vaccine compliance was thus conceived as a means to achieve herd immunity. However, with the arrival of the Omicron variant, it became apparent that the vaccine was still a helpful tool against the more severe forms of the disease but was much less effective against its transmission. In light of this, the use of more intrusive instruments to prompt vaccination (the enhanced Green Pass and the mandate for the over-50 s) seems to serve a different purpose than the original design, namely to reduce the pressure of COVID patients on hospitals and the health service in general. This explains why the over 50 s were targeted by the most restrictive measures even though the percentage of unvaccinated was higher among the younger cohorts. From this point of view, it seems as if the Italian vaccination campaign, particularly in the last phase, ran on a separate track from the other instruments that could be used to keep infections under control, about which the government has long held a less rigid stance. For example, the obligation to wear ffp2 masks indoor and on public transport was only introduced with decree no. 221 at the end of December 2021, when the wave of new infections was out of control; likewise, for workers under 50, who could keep going on with basic Green Pass to access workplaces, it was still possible to prove virus negativity with rapid swabs at reduced prices, which were much less reliable than molecular tests. In a sense, therefore, the Italian government seems to have been so focused on recalibrating the instrument it had relied on to make the vaccination campaign work, that it left behind the issue of integrating the instrument mix developed to fight the virus.

## Concluding remarks: a pragmatic road to compliance?

Should we have to use a single word to encapsulate the Italian government's strategy about the vaccination campaign against COVID-19, it would undoubtedly be 'pragmatism'. According to the distinction originally made by Ansell and Boin (2019), and later applied by Boin and Lodge (2021) to the COVID-19 emergency response, a pragmatic approach to handle crisis-driven uncertainty differs from a principled approach in that it gives preference to incremental trial and error strategies of adaptation over the making of ‘do or die’ decisions. Indeed, while the Italian government basically adhered to the golden principle of the least restrictive alternatives, its timing in scaling up the intrusiveness scale, as well as the type of instruments selected each time and combined with the ones already in place, have followed an incremental trial-and-error approach, at the same time discarding from the available pool of instruments those never tried in the past, whose acceptance by the target audience might have been uncertain. A glaring example of this strategy is the Green Pass, an instrument that has undergone an outright *conversion* process (Streeck & Thelen, 2005) over the months: while retaining the same name, and thus exploiting a kind of ‘habit effect’ on the recipients, it has gradually shifted from the category of incentives or nudges to coercion, especially when its enhanced version was demanded for access to the workplace (Palmieri & Goffin, 2022). At the same time, in climbing the ladder of intrusiveness, the government took into account both the political and technical feasibility of the instruments on the table, only reaching the highest stage of coercion (the compulsory vaccination for the over-50 s) when there was the opportunity to seize the moment: contagions were growing rapidly, polls were in favor of more restrictive measures, and other European governments had already paved the way (although in some cases, as in Austria, mandatory vaccination was limited to announcements without actually enforcing it).

All in all, such a pragmatic approach—based on the incremental and selective layering of policy instruments (Boin & Lodge, 2021, 1137)—certainly brought some advantages: at the end of the day, with regard to effectiveness, the vaccination coverage achieved in Italy was among the highest in Europe^16^; the protests of the Italian anti-vaccine and anti-mandatory movements have been less vehement and less violent than in other countries (like France), and have faded over time; and, last but not least, Prime Minister Draghi avoided irreparable rifts in his large but fractious government coalition, whose cohesion was as yet undermined by the many and pressing commitments on the post-pandemic recovery front—not least those related to the management of the National Recovery and Resilience Plan (Di Mascio et al., 2022).

Some flaws in this strategy were nevertheless becoming apparent when, to urge the most resistant people to vaccinate, the government moved to the top rung of the ladder. Since then, the instrument mix has lost some of its internal coherence; its fit with the goal became less apparent and persuasive; and even the real effectiveness of the most intrusive measures for the over-50 s, according to available figures, seems not so striking.

Shortly after the introduction of the vaccination mandate, due both to the gradual decline in the number of infections and to the onset of other emergencies such as the Russian-Ukrainian conflict, the attention (both of the government and of the public) was noticeably drawn away from the vaccination campaign, and it is therefore difficult to assess whether and to what extent the aforementioned flaws had an impact on the perceived legitimacy of government's strategy. The strengths and weaknesses highlighted in this paper do, however, offer a starting point for future studies—including comparative research—on how, under what conditions and with what effects governments assemble their vaccination policy toolbox.

## Footnotes

1 See “French to get access to Covid jabs on holiday including at the beach”, *The Guardian*, 5 July 2021.

2 See “Uber, Bolt and Deliveroo help drive vaccine uptake”, Press Release on https://www.gov.uk, 1 August 2021.

3 In particular Tuscany, Campania, Sicily and the Autonomous Province of Bolzano.

4 Report Vaccini Anti-COVID—Governo Italiano, April 2, 2021.

5 “Draghi: ‘Senza vaccinazione si deve chiudere tutto di nuovo’”, *Il Sole 24 ore*, 22 July 2021.

6 See “Vaccini, boom di prenotazioni nelle Regioni. Figliuolo: da + 15% a + 200%”, *Il Sole 24 ore*, 23 July 2021.

7 See “Vaccini e Green pass: la protesta nelle piazze d'Italia”, *La Repubblica*, 24 July 2021.

8 See “Vaccini, i numeri aggiornati sul personale della scuola”, *Il Sole 24 ore*, 20 August 2021.

9 GIMBE Foundation press release, 29 July 2021.

10 See “Vaccini, boom di prime dosi tra i cinquantenni: + 52%”, *Il Sole 24 ore*, 28 September 2021.

11 GIMBE Foundation press release, 18 November 2021.

12 Ibidem.

13 “Obbligo vaccinale over 50: dopo un mese uno su 3 ha «ceduto»”, *Il Sole 24 ore*, 5 February 2022.

14 More precisely: 58% on 4 April (Demos), 65% on 7 September and 64% on 13 December 2021 (Ipsos).

15 GIMBE Foundation press release, 17 March 2022 (available at https://www.gimbe.org/pagine/341/it/comunicati-stampa?pagina=18).

16 https://vaccinetracker.ecdc.europa.eu/public/extensions/covid-19/vaccine-tracker.html#uptake-tab (Accessed: 23 May 2022); https://covid19.who.int/table (Accessed: 23 May 2022).
